# Socioeconomic and Geographic Pattern of Food Consumption and Dietary Diversity among Children Aged 6–23 Months Old in Ghana

**DOI:** 10.3390/nu13020603

**Published:** 2021-02-12

**Authors:** Isaac Anane, Fengying Nie, Jiaqi Huang

**Affiliations:** 1 Agricultural Information Institute of Chinese Academy of Agricultural Sciences, Beijing 100081, China; 2019Y90100053@caas.cn (I.A.); huangjiaqi@caas.cn (J.H.); 2 Urban Economics Group, Department of Social Sciences, Wageningen University, 6706 KN Wageningen, The Netherlands

**Keywords:** dietary diversity, socioeconomic status, geographical locations, food consumption, Ghana

## Abstract

Dietary inadequacy is a major challenge among young children in Ghana. Nutritional policies are required for optimum child nutrition and development. This study explored food consumption and dietary diversity by socioeconomic status and geographical location among children aged 6–23 months in Ghana. We used the latest national representative, cross-sectional data from the Ghana Demographic and Health Survey (GDHS-2014). A total of 887 children aged 6–23 months were used in the final analysis. The survey collected data on children’s food consumption through their mothers in the 24 h recall method. Multiple logistic regression models were used to assess the relationship between socioeconomic status and geographical location with food consumption and adequate dietary diversity after adjusting for control variables. The study revealed an association between specific food item consumption, food groups, and dietary diversity by socioeconomic and geographic characteristics. However, dairy consumption increased faster than other nutritional foods when socioeconomic status increased. Furthermore, the study revealed that children’s chances of consuming particular food items and food groups differed across Ghana’s 10 regions. The average probabilities of consuming adequate dietary diversity between the Greater Accra region and Ashanti region were 43% vs. 8% (*p* < 0.001). Consumption of grains, root, and tubers were relatively higher but low for Vitamin A-rich fruits and vegetables and legumes and nuts for children aged 6–23 months in Ghana. Overall, the mean dietary diversity score was low (3.39; 95% CI: 3.30–3.49) out of eight food groups, and the prevalence of adequate dietary diversity was 22% only. There is a need for policy interventions to ensure appropriate dietary practices to promote healthy growth of children.

## 1. Introduction

The health state is greatly influenced by the food intake and utilization of food nutrients [1,2]. During the early years, poor diet contributes to severe malfunctions, including poor academic performance, poor social skills, delay in motor and cognitive growth, and behavioral disorders [3]. Therefore, it is undeniable that infants and young children require optimum nutrition and acceptable feeding practices for optimal growth, healthy life, and cognitive development [4,5]. Globally, sustainable development goals (SDGs) statistics show that in the year 2017, about 821 million were undernourished, and about 45% of deaths recorded each year among children less than five years were caused by poor diet [6,7,8]. Worldwide, it was estimated in 2018 that 149 million or 22% of children under five years were still chronically undernourished, and 36% of the world’s chronically malnourished lived in sub-Saharan Africa [7].

Ghana’s undernutrition has improved steadily over the decades; however, child malnutrition is a major public health problem [9,10]. The anemia rate among children countrywide remains high (66%) due to micronutrient deficiencies [11]. It is estimated that in 2008 the prevalence of stunting, underweight, and wasting among children under five years were 28%, 14%, and 9%, respectively [12]. Poor diet remains high in the rural communities, and many of the problems are deeply rooted in limited knowledge of infant and young child feeding practices, poverty, and poor food distribution networks [13]. In a broad category, about 5% of Ghana’s population is estimated to be food insecure, and about two million people are susceptible to becoming food insecure. The government of Ghana and stakeholders have set up the National Nutrition Policy (NNP) to ensure optimal nutrition. The policy managers are tasked with enhancing the consumption of diverse diets and promoting nutrition intake by women of child-bearing age and newborns. The economic losses poor nutrition brings to an economy are enormous, and it is vital to encourage investment in the social sector, particularly nutrition policies for young children, to improve social and economic development.

One of the underlying causes of child malnutrition is inadequate nutritional intake [14,15]. It has been documented that at least one in three children under five years old in the world is not growing well because of malnutrition in a more noticeable form, such as stunting, wasting, and overweight [16]. Malnutrition is closely linked to household food insecurity, young children and infant feeding practices, and socioeconomic status (SES) [17,18]. Socioeconomic factors such as household wealth status, parents’ educational level, and geographical location significantly influence a child’s nutritional status [19,20].

Most researches on child malnutrition apply anthropometric failures such as stunting, wasting, and underweight rather than dietary diversity in their analysis [8,15,17,18]. While dietary diversity and anthropometric failures are interconnected, it is essential to emphasize dietary diversity as many nutritional intervention programs aim to improve food quantity and quality [21,22]. Previous research has established that dietary diversity positively impacts micronutrient adequacy and improves young children’s nutritional status [23,24,25]. In other words, low dietary diversity is associated with stunting, underweight and wasting among young children and affects overall child development [26,27]. Dietary diversity is an easy and reliable approach to conceptualize young children’s feeding practices and adequate nutritional consumption [28,29]. Many proponents have stated that socioeconomic and demographic characteristics strongly correlate with dietary diversity [24,30,31]. Maternal education, household wealth status, geographical area, and other societal norms affect children’s feeding practices and dietary diversity. It has been established that children from wealthy households have access to diverse foods and a higher level of maternal education turns out to have a positive relationship with dietary diversity [32,33].

Previous studies have established the relationship between child dietary diversity and socioeconomic and demographic characteristics [34,35,36,37,38,39,40]. A few studies have been conducted on a child’s nutritional adequacy using nationally representative data in Ghana [36,37,38]. Perhaps, due to data limitation, most existing research in Ghana focuses on particular geographical locations and leaves out places where children suffer from malnutrition [34,36,37,38]. Another concern is about the paucity of research on child dietary diversity and socioeconomic characteristics among children aged 6–23 months in Ghana. Most of the research investigating the relationship between dietary diversity and socioeconomic factors was based on children aged 6–59 months old [35,40]. In the early years of life, good nutrition is essential because body growth and brain development are faster than at any other time [41,42]. The failure to optimize good nutrition has a long-term consequence on job potential, education, and adult mental health [43]. The minimum dietary diversity among children aged 6–23 months in Ghana is still below the World Health Organization (WHO) recommendation [44]. The importance of early years of good nutrition combined with inadequate minimum dietary intake in Ghana make it crucial to conduct thorough studies among children aged 6–23 months. Besides, the studies have made an effort to include specific food items in the analysis that most of the previous work conducted in Ghana failed to include [44,45,46,47]. This study included an age group that has not been extensively studied and relied on nationally representative data. The study aimed to promote nutritional policies to enhance dietary diversity among children aged 6–23 months in Ghana through food quality and quantity.

This present study used the most recent nationally representative data in Ghana to explore the socioeconomic status and geographical location pattern of specific food item intake, food groups’ consumption, and dietary diversity adequacy among 6–23-month-old children.

### Conceptual Framework

Several models explain the determinants of a child’s nutritional status. This paper’s conceptual framework was adapted from the United Nations Children’s Fund (UNICEF) framework for the causes of malnutrition. The framework demonstrates that both biological and socioeconomic factors cause children’s malnutrition. The framework categorizes the determinant factors into three main groups: immediate, underlying, and basic. The immediate determinants happen at the individual level and describe dietary intake and health status. The underlying determinants take place at the household level. The underlying causes include food security, mother and children care practices and proper health environment. The basic causes manifest at the societal level. The basic level determinants concern the potential resources available to a country, technological accessibility, and human resource quality. The framework suggests that politics, cultural, economic, and social systems, including women’s status in society, affect potential resource utilization. The determinant factors are interconnected, and one level influences the other level.

**Figure 1:**
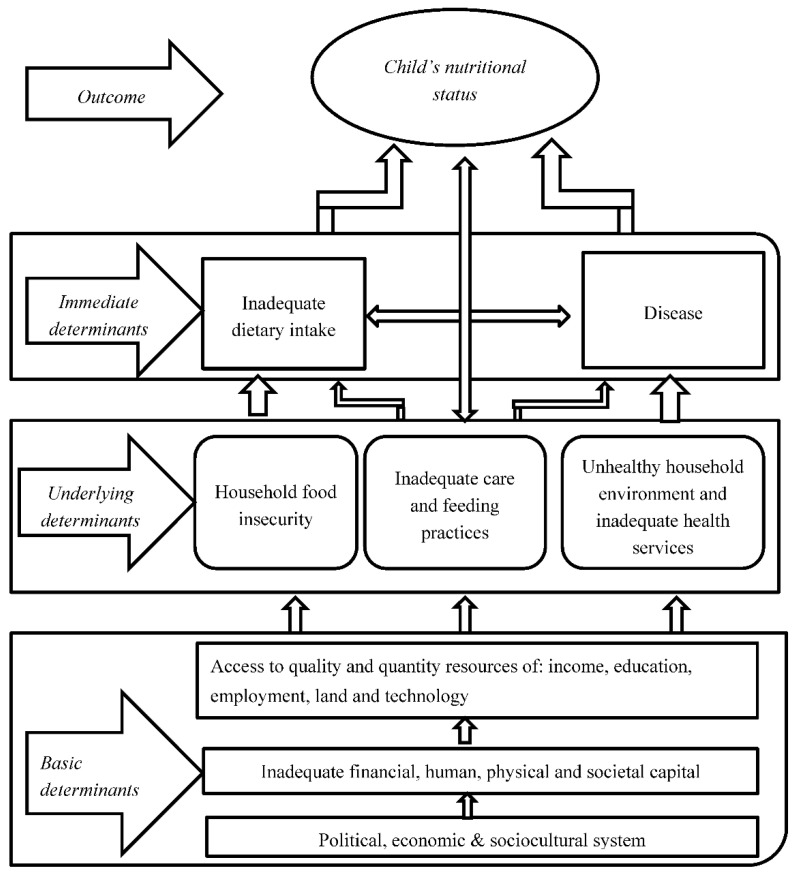
Conceptual framework: the determinants of child nutritional status. Source: Adapted from UNICEF 1998, 2015 [14,53].

Proponents of this framework posit that once a child lives in a community, changes in the community can consciously or unconsciously affect their nutritional status through environmental, socio-demographic, and individual factors. Children from a high socioeconomic background are likely to have healthier food habits and good dietary profile consistency with dietary guidelines, hence good nutritional status [48,49,50]. The framework explains how socioeconomic factors function to impact the nutritional quality through intermediate and proximal variables. High dietary diversity enhances absorption of energy and nutrients, dietary adequacy, and child development and nutrition [38]. According to the framework, the basic determinants act through underlying factors, immediate factors, and proximal factors to affect child nutrition. For instance, socio-demographic characteristics such as mothers’ education, household wealth, and geographical location may directly or indirectly affect child care and feeding practices and dietary intake. Many relevant studies use wasting, stunting, and underweight to measure child nutrition [8,15]. This study used adequate dietary diversity as a proxy to measure child nutrition adequacy, given the fact that many children’s nutritional interventions seek to improve child growth through food quantity and quality [21]. Based on the interrelationship of the previous relevant literature and the framework, variables were derived from measuring the child’s nutrition [38,45,51,52] (Figure 1). The specific variables included in the logistic model to achieve the research objective included the child’s age, gender, birth order, mother’s education, household wealth, geographical location, ethnicity, religion.

## 2. Materials and Methods

### 2.1. Study Design and Data

This study used the latest and nationally representative cross-sectional data set from the Ghana Demographic and Health Survey (GDHS) in 2014 [54]. The data set was obtained from the website of Measure DHS. The data set was exempted from specific permission because it was based on an anonymous public use data set with no identifiable information of the study population. Permission to the data set was given after the application to use the data was accepted [55]. GDHS-2014 was selected for this study because it is the most recently released data set and provides detailed information on dietary diversity and nutritional variables. The GDHS-2014 was administered by the Ghana Statistical Service (GSS) and Ghana Health Service (GHS) with technical and financial assistance from International Classification of Functioning, Disability and Health (ICF) International and the United States Agency for International Development (USAID), respectively. The GDHS-2014 used a two-stage sample design, and it was structured to include key national-level indicators, urban and rural, and each of Ghana’s 10 administrative regions. The first stage adopted sample points (clusters) from enumeration areas (EAs) designed for the 2010 Population and Housing Census (PHC). The first stage included 427 clusters, of which 211 were in rural areas and 216 were in urban areas. In the second stage, the households were selected through a systematic sampling technique from these clusters. In all, about 30 households were selected from each cluster. In total, 12,831 households were selected for the survey, of which 12,010 were occupied. A total of 11,835 households were successfully interviewed out of the 12,010 occupied households, yielding a response rate of 99% [56]. All men and women aged 15–59 and 15–49, respectively, were qualified to join the survey.

**Figure 2:**
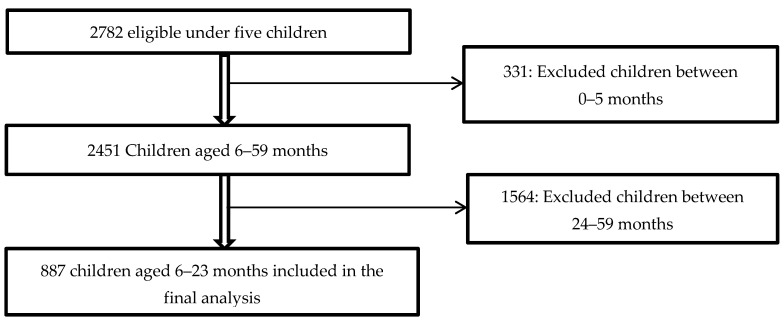
Participant flow chart.

The GDHS-2014 used three sets of questionnaires: The Household Questionnaire, the Woman’s Questionnaire, and Man’s Questionnaire. In the standard women’s questionnaire, a total of 9656 women were qualified to participate, of which 9396 were successfully interviewed, yielding a response rate of 97% [56]. The women’s questionnaire included a child health section. The child health component included only women with at least one child under five years before the survey, and consisted of 2782 respondents. This study limited the study population to young infants and children aged 6–23 months. The sample size included in the final analysis was 887 children. A detail of participant flow in this analysis is depicted in Figure 2.

### 2.2. Ethical Statement

The 2014 Ghana Demographic and Health Survey (GDHS-2014) was conducted by the Ghana Statistical Service (GSS) in collaboration with the Ghana Health Service (GHS) and National Public Health Reference Laboratory (NPHRL). The GSS, GHS, and NPHRL received technical assistance from the International Classification of Functioning, Disability and Health (ICF) through DHS programs [54]. The ICF Macro Institutional Review Board (IRB) and the Ghana Health Service Ethics Review Committee received ethical clearance from the National Public Health Reference Laboratory of the GHS and Noguchi Memorial Institute for Medical Research all in Ghana before GDHS-14 was carried out [57]. The ethical committees received written informed consent from the study participants before the interview. Also, this paper’s authors requested and received permission from the DHS to use the data. The authors of this paper needed no more ethical approval to use the completely anonymous public data set.

### 2.3. Description of Variables

#### 2.3.1. Outcome Variable

The main outcome variable for this study was adequate dietary diversity consumption (ADDC). The ADDC was used as a dependent variable. The study’s three key independent variables were household wealth, mothers’ education and geographical location, and other controlled confounders. However, specific food items and food groups were also used as dependent variables to show the pattern of consumption of particular food items or food group changes when socio-demographic characteristics change. The ADDC was created based on the mother’s recall of the child’s consumption of food groups over 24 h immediately preceding the mother’s interview. Data were gathered on the number of specific foods consumed from the previous day: juice; tinned powdered/fresh milk; fortified baby food; other liquids; bread, noodles, other grains; potatoes, cassava, tubers; eggs; pumpkin, carrot, squash; dark green leafy vegetables; fruits; meat (chicken, beef); liver, heart, other organ meat; fish, shellfish; beans, peas, lentils, and other solid/semi-liquid food. The food items were categorized into eight food groups recommended by WHO and the WHO-UNICEF Technical Expert Advisory Group on Nutrition Monitoring (TEAM) [28,58,59]. In 2017, the WHO changed the food groupings from seven to eight food groups to eliminate the differences in breastfeeding indicators compared with non-breastfeeding children [60,61]. Details of food item groupings are shown in Table 1 below. A dietary diversity score was generated by gathering information on food groups. The food group was given a range of 0–8. A score of 1 was assigned to children who consumed at least one of the food groups and 0 for children who did not consume food items from the food groups. A binary variable was generated from the total dietary diversity score to get adequate dietary diversity consumption (ADDC). Children were considered to have ADDC if they had consumed five or more food groups, whereas children who consumed four or fewer of the food groups were considered inadequate [60].

#### 2.3.2. Main Independent Variables

The various socio-demographic factors that were used included maternal and child demographic factors, household factors, and community characteristics. Three child demographic characteristics were used in the analysis. They were the child’s age, and child’s gender, and birth order. The maternal demographic factors included were education, ethnicity, and religion. Education level was divided into four categories: no education, primary (1–5 years of education), secondary education (6–8 years of education), and higher education (9 years and above of education). Religion fell into four different categories: Christianity, Islamic, African Traditional Religion, and other/No religion. Ethnicity was categorized into nine different groups: Akan, Ga/Dangme, Ewe, Guan, Mole–Dagban, Grusi, Gurma, Mande, and Other.

The household-level factor included in the analysis was the wealth index. The household wealth index is a composite measure of the socioeconomic position [61,62]. The household wealth index is computed based on households’ ownership of assets and consumer items such as refrigerators, washing machines, bicycles, and access to water and sanitation facilities through principal component analysis [63,64,65]. Each index had a mean value of 0 and a standard deviation of 1, and it was divided into five quintiles: poorest, poorer, middle, richer, and richest [51].

Community-level characteristics included were geographical location. Ghana is divided into 10 administrative regions as of 2014, and each region faces different challenges in food accessibility. The region categorization includes Western, Central, Greater Accra, Volta, Eastern, Ashanti, Bono-Ahafo, Northern, Upper East, and Upper West.

### 2.4. Statistical Analysis

All statistical analysis was done using Stata 16 (M.P.—Parallel Edition). Sample weights were calculated based on the sample’s non-proportional distribution to the different survey clusters and residents (rural and urban). The sample weight was done purposely to ensure that our analysis was nationally representative and adequately distributed to all different survey clusters.

A set of logistic regression models was applied to estimate the odds ratios (ORs) of mothers’ education and household wealth on adequate dietary diversity consumption (ADDC), food groups, and specific food items. Model A displayed the relationship between mother’s education, household wealth, and each result after adjusting only the child characteristics (i.e., age and gender), while Model B further controlled for residence, religion, ethnicity, child’s birth order. Afterward, a set of multiple logistic regression models was used to estimate the Average Predictive Margins (APMs) of geographical location on the consumption of specific food items, food groups, and ADDC with adjusted variables such as child’s age in months, child’s gender, child’s birth order, mother’s education, ethnicity, and religion.

Many researchers use coefficients, *p*-values, standard errors, confidence intervals, or significance stars to communicate their statistical model results. However, there are some limits and interpretational difficulties, especially when it comes to categorical variables and nonlinear models such as logistic regression [66,67]. The use of APMs makes the results from the analysis much more meaningful, intuitive, and easy to interpret [66,67,68,69]. Hence, the study used APMs to predict children’s average probability of having ADDC based on the geographical location.

The study conducted two sensitivity analyses to check the models’ robustness and how changes in other variables affected the outcome. Given that, nutritional problems also exist among older children. The first sensitivity analysis was conducted among older children aged 24–59 months with dietary data. Furthermore, the study evaluated whether, after adjusting for the state’s fixed effects, they would significantly change our analysis. All the underlying multivariable regressions were tested to know the possibility of multicollinearity in work. A correlation matrix of covariates was used to assess the likelihood of multicollinearity. However, all pairwise correlation coefficients were less than 0.5, indicating an absence of multicollinearity [70].

## 3. Results

### 3.1. Specific Food Items, Food Groups, Dietary Diversity, and Socio-Demographic Information of the Study Population

Table 2 shows the frequency and percentages of young children aged 6–23 months by selected socio-demographic factors. The results revealed that about half of the sampled children were male. Forty-nine percent of the mothers received secondary education, while 28% received no formal education. A majority of the mothers (48%) belonged to the Akan ethnic group. Seventeen percent of the mothers lived in the Ashanti region, and more than half (76%) of the mothers were Christians. Almost a quarter (23%) of the sample households belonged to the poorest quintile.

Table 3 shows the frequency and percentages of young children aged 6–23 months who consumed specific food items and food groups. Regarding the children who consumed any of the 18 food items, 81% had bread, noodles, and other grains. Around 45% consumed fish, shellfish (45%), dark green leafy vegetables (36%), other solid/semi-liquid food (36%); potatoes, cassava, tubers (30%); eggs (20%); fortified baby food (16%); tinned powdered/fresh milk (15%); meat (chicken, beef) (13%); beans, peas, lentils (13%); juice (13%); other liquids (9%); pumpkin, carrot, squash (7%); fruits (25%) and liver, heart, other organ meat (3%) in the last 24 h. The findings revealed that, among the eight food groups, most of the children consumed grains, roots, and tubers (89%), followed by breast milk (84%), flesh foods (52%), other fruits and vegetables (47%), dairy products (22%), eggs (20%), legumes and nuts (13%) and Vitamin A-rich fruits and vegetables (12%) in the previous 24 h (Table 3). Overall mean dietary diversity score was low (3.39; 95% CI: 3.30–3.49) out of eight food groups. Adequate dietary diversity consumption (ADDC) was 22%, while inadequate consumption was 78%.

### 3.2. Average Consumption of Specific Food Items, Food Groups, and Dietary Diversity Consumption by Socio-Demographic Characteristics

Table 4 and Table 5 present the consumption of particular food items, food groups, and adequate dietary diversity intake measured by the mother’s education, household wealth, and geographical location among children aged 6–23 months old. The findings revealed a significant difference (*p* < 0.001) in consumption of food items such as juice, tinned powdered/fresh milk, fortified baby food, eggs, and fruits by the mother’s education (Table 4). For instance, the intake of juice (9% vs. 37%), tinned powdered/fresh milk (6% vs. 50%), fortified baby food (4% vs. 66%), eggs (13% vs. 59%) was four times or more higher for mothers with the higher educational background than mothers with no formal education. Threefold differentials were observed in the consumption of fruits (20% vs. 60%) between mothers with higher education and those with no education. Among the food groups, the highest differentials were found in the intake of breast milk (89% vs. 69%), followed by dairy (11% vs. 63%) and eggs (13% vs. 59%) by the mother’s education. Children whose mothers had higher educational backgrounds consumed less breast milk than mothers with no academic background. Mothers with higher educational backgrounds had a mean dietary diversity score of 4.8, compared to mothers with no education at 3.3. Children of mothers with higher educational backgrounds had an adequate dietary diversity consumption of 65%, compared to 19% for those of mothers with no education.

The study also found a considerable difference (*p* < 0.001) in the consumption of numerous food items by household wealth among children aged 6–23 months old (Table 4). For instance, children from the wealthiest households had four times or more differentials than the children from the poorest households in the consumption of food items such as juice (6% vs. 25%), fortified baby food (8% vs. 41%), eggs (6% vs. 33%), and tinned powdered/fresh milk (6% vs. 34%). Again, a threefold differential was found in the consumption of fruits (12% vs. 37%) between children from the richest households and poorest households. Regarding the food groups, a substantial differential by household wealth was found in the consumption of dairy products (6% vs. 52%) and eggs (6% vs. 33%). Here, children from the wealthiest households had higher percentages in the consumption of dairy products and eggs than children from the poorest households. The mean dietary diversity score ranged from 3.0–4.0 among children from the poorest to the richest households. Children from the richest households had an adequate dietary diversity of 38%, compared to 13% for those from the poorest households.

The study also revealed a substantial difference in the consumption of several food items by the 10 regions in Ghana. For example, children who lived in Greater Accra were found to consume four times or more food items, such as juice (27% vs. 3%), tinned powdered/fresh milk (28% vs. 2%), fortified baby food (22% vs. 3%), meat—chicken, beef (30% vs. 8%), than their counterparts who lived in the Upper West (Table 5). The percentage of children from the Western region who consumed juice (19% vs. 3%), fortified baby food (23% vs. 2%), and eggs (38% vs. 8%) (Table 5) exceeded by four times the percentage of children from the Northern region. Regarding the food groups, the largest differential by region was found for the consumption of dairy products (55% vs. 4%), with children from the Greater Accra region consuming more than children from the Upper West. The mean dietary diversity across the 10 administrative regions ranged from 2.7 to 4.5, with the highest recorded in Greater Accra and the least recorded in the Eastern region. However, the highest adequate dietary diversity consumption was recorded in Greater Accra, while the least was recorded in the Ashanti region.

### 3.3. Logistic Regression Model (ORs with 95% CI) for an Association between Specific Food Items, Food Groups, ADDC, and Socioeconomic Characteristics

Table 6 displays two-part model odds ratios (ORs) and 95% confidence intervals (CIs) of household wealth on the consumption of specific food items and food groups of children aged 6–23 months. Multivariate logistic regression results are presented in Table 6 with ADDC, specific food items, and food groups as dependent variables, household wealth as the main independent variable, and adjusted other covariates. Model A adjusted for only child characteristics (age and gender), while Model B adjusted for full factors such as child, maternal, household, and community-based characteristics. The study finding from Model A showed those children from the richest households had four times or more odds ratios than children from the poorer households in consuming food items such as juice, tinned powdered/fresh milk, and fortified baby food. Again, children from the richest households consumed two or more times the quantities of food items such as eggs and pumpkin, carrot, squash. The odds ratio was further confirmed after adjusting all characteristics (Model B). The study revealed that children from the wealthiest households were more likely to consume more of the food items such as juice, tinned powdered/fresh milk, fortified baby food, bread, noodles, other grains, fruits, eggs, other solid/semi-liquid food, liver, heart, other organ meat and pumpkin, carrot, squash than children from the poorer households. Regarding the food groups, dairy product consumption was four times or higher among children from the richest households than children from the poorer households in each separate model. On the whole, the study found that children from the poorest households had a lower odds ratio of adequate dietary diversity intake, compared to children from the wealthiest households, with ORs of 1.25 (95% CI: 0.75, 2.07) and 1.13 (95% CI: 0.64, 2.00) in Model A and B, respectively (Table 6).

Table 7 presents ORs of children aged 6–23 months with CIs of mothers’ education on consuming specific food items and food groups in the two-part model. Multivariate logistic regression results are presented in Table 7 with specific food items, food groups, and ADDC as dependable variables and mother’s education as the main independent variable and controlled for other covariates. Mothers with higher educational backgrounds had higher odds than mothers with no formal education. For example, food items such as juice, tinned powdered/fresh milk, fortified baby food, eggs and liver, heart, other organ meat consumption were higher for high education-level mothers than for no education mothers (Model A). The same relationship trend was found after fully adjusting the model (Model B). The study revealed a higher odds ratio among children whose mothers had a higher education on consuming specific food items such as tinned powdered/fresh milk, fortified baby food, juice, liver, heart, other organ meat, and eggs than children whose mothers had no education.

Regarding the food, the largest significant odds ratio was observed among children who had mothers with a higher educational background in the consumption of dairy products, followed by eggs (Model A). The same relationship trend was observed after adjusting all other characteristics in Model B. Overall, children who had mothers with higher educational backgrounds had higher odds ratios of adequate dietary diversity intake than children whose mothers had no education, with an OR of 3.52 (95% CI: 1.58–7.85) in Model A and 2.18 (95% CI: 0.80–5.92) in Model B.

### 3.4. Average Predictive Margins (APMs) for an Association between Specific Food Consumption, Food Groups, ADDC, and Geographical Locations

Table 8 presents the Average Predictive Margins (APMs) with standard error (SE) for a geographical area on consuming specific food items and food groups among children aged 6–23 months. Multivariate logistic regression results are presented in Table 8 with specific food items, food groups, and ADDC as dependable variables and geographical location as a main independent variable, and adjusted other confounders. APMs were set up to predict the probability of consuming particular food items and food groups by the child’s location in any of Ghana’s 10 administrative regions. The analysis showed that the likelihood of children consuming specific food items and food groups differed among Ghana’s 10 regions. For example, on average, the probability of children located in Greater Accra to consume food items such as juice was 0.20 or 20%, tinned powdered/fresh milk, compared to a child from Upper West at 0.04 or 4%. The same trend was observed among food items such as tinned powdered/fresh milk (16%), fortified baby food (18%) for children from the Greater Accra region, compared to tinned powdered/fresh milk (4%), fortified baby food (4%). Again the Western region and Northern regions give different average probabilities of food consumption. For instance, children from the Western region had average predicted probabilities in the consumption of food items such as juice, fortified baby food, eggs of 21%, 22%, 37%, respectively, compared to children from the Northern region of juice (3%), fortified baby food (4%), and eggs (9%) (Table 8).

Regarding the food groups, children located in Greater Accra had a predictive margin of 34% in dairy product consumption compared to that of children from the Upper West of 8% in dairy product consumption. A higher variation of predictive margins was observed in egg consumption between the Western region (37%) and Upper East (9%).

**Figure 3:**
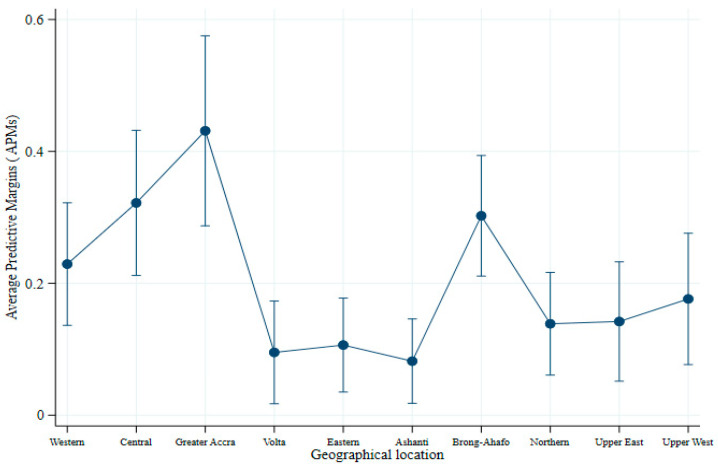
Average predictive margin of geographical location with 95% CIs.

Overall, a higher magnitude of average predictive margin was observed between geographical areas after adjusting child, maternal, household, and community characteristics. The findings revealed that the probability of a child having an ADDC was 0.43 or 43% in Greater Accra, compared to children from Volta (10%) and Ashanti (8%) (Table 8). The highest average predictive margin was recorded in the Greater Accra region, followed by Central, Bono-Ahafo, Western, Northern and Upper East, Upper West, Eastern, Volta, and Ashanti, in that order (Figure 3).

### 3.5. Sensitivity Analysis

The sensitivity analysis conducted among children aged 24–59 months old revealed a lower mean dietary diversity intake of 2.63 (Appendix A Table A1) than children aged 6–23 months (3.39). The analysis revealed the same trend in consuming specific food items, food groups, and adequate dietary diversity consumption among these age groups. The study found no interaction between predictor variables maternal education, household wealth, and geographical area, and ADDC among children aged 6–23 months (*p* = 0.305). Moreover, clarifying with states’ fixed effects further confirmed a similar relationship between mother’s education, household wealth, and ADDC. For example, the OR of household wealth between poorer and wealthiest households were 0.96; 95% CI: 0.52–1.78 and 1.90; 95% CI: 0.75–4.85, respectively. Also, the ORs of mothers’ education between high educational background mothers and no education mothers were 2.22; 95% CI: 0.82–6.07 and 0.82; 95% CI: 0.46–1.49 (Appendix A Table A2). The sensitivity analysis confirmed that children from wealthy backgrounds had higher ADDC odds than children from poor households, while children whose mothers had higher education levels also had higher odds than children whose mothers had no education.

## 4. Discussion

This study was set up to explore the pattern of specific food consumption, food groups, and ADDC among 6–23-month-old children in Ghana. Our analysis showed that ADDC was significantly associated with socio-demographic characteristics. The relationship between ADDC and socio-demographic characteristics, including maternal education, household wealth, and geographical locations, remained significant after adjusting other covariates. This study’s findings were consistent with previous work indicating an association between adequate dietary diversity score and socioeconomic status [31,71,72]. This work’s results were consistent with a study conducted in Ghana that found a positive relationship of maternal education and household wealth with a child’s dietary diversity score [38,40,45]. The study further revealed large disparities in the consumption of specific food items, food groups, and dietary diversity across Ghana’s 10 administrative regions among children aged 6–23 months. The findings further confirmed that food accessibility challenges transition into Ghana’s dietary diversity problem [73,74]. Children from certain regions, particularly Greater Accra, Western, Bono-Ahafo, and Central region, were more likely to have higher chances of consuming an adequate dietary diversity than their counterparts from the Volta, Eastern, and Ashanti regions. These findings were consistent with several studies conducted in Ghana and other developing countries that found differences in national nutrition adequacy among children in different locations [44,47,75,76]. Agriculture is mainly rain-fed in Ghana, and farmers face challenges from climate change, the poor road network, inadequate marketing, and lack of access to finance [77]. Climate conditions have a huge impact on food production in all parts of the country, together with other problems such as the poor road network, lack of finance, and inadequate market results in food security problems in different regions of the country.

Mainly, food items and food groups such as juice, tinned powdered/fresh milk, fortified baby food, eggs, and fruits were strongly associated with maternal education and household wealth. The findings revealed that maternal education and household wealth are relevant factors that determine food varieties’ consumption. This finding was consistent with similar research conducted in Rwanda and research to determine factors that influence children’s eating behaviors [78,79]. The multivariate analysis of those studies showed that consumption of a variety of food depends on maternal education and household wealth. Overall dietary diversity intake was found to increase with household wealth and mother’s education. However, most of the higher differentials were observed among the consumption of dairy products. For example, higher differential was found in the consumption of tinned powdered/fresh milk (6% vs. 50%) by mother’s education and (4% vs. 25%) by household wealth. Meanwhile, a small differential was observed in high protein source foods such as fish and shellfish, with a higher differential for poorer vs. richest (38% vs. 47%) and higher education vs. no education (41% vs. 56%).

Furthermore, the study revealed that food items and food groups’ consumption was strongly correlated with the children’s geographical location. The finding was consistent with other previous studies [80,81,82]. The previous work’s findings revealed that globally, geographical location affects dietary consumption due to food availability and affordability constraints. Great discrepancies in ADDC among children aged 6–23 months were observed in Ghana’s 10 administrative regions, with a magnitude of 0.43 or 43% and 0.08 or 8% between the highest and lowest.

Generally, the consumption of carbohydrate foods (grains, root, and tubers) across the 10 regions was relatively higher than Vitamin A-rich fruits and vegetables. For example, the average intake of grain, root, and tubers in Greater Accra was 94% compared to Vitamin A-rich fruits and vegetables at 66% (Table 6). Further studies to establish the trend of carbohydrate and vitamin intake across Ghana’s regions will help.

The study has both strengths and limitations. The important strength is using nationally representative data from GDHS to analyze the dietary diversity among children aged 6–23 months in Ghana. Again, the use of the eight food groups recommended by WHO-IYCF in 2017 adds more advantages [59]. Moreover, the 24 h recall method in dietary data collection used by the DHS is almost the shortest recall time and is considered more accurate than the longer period because it reduces recall bias chances [77]. However, the use of a cross-sectional design of the survey limits the assessment of causality link. Although 24 h recall is considered the shortest recall time and reduces participants’ memory burden, recall bias may be present as participants can be selective with the foods they choose to recall [83]. Furthermore, the lack of detailed information on important variables such as the cooking method used, seasonality data, and portion size can be a limitation. Specific food consumption varies across seasons. Regardless of all the limitations, the study provides a comprehensive and consistent relationship in all three stated variables.

## 5. Conclusions

Dietary inadequacy is a major challenge among young children in Ghana. The high level of food insecurity makes it difficult to ensure dietary diversity among children aged 6–23 months. This study aimed to explore the relationship of socioeconomic status and geographical location with ADDC, specific food items, and food groups among children aged 6–23 months in Ghana. The research showed an association of the consumption of specific food items, food groups, and dietary diversity with maternal education, household wealth, and geographical location. However, dairy product consumption increased faster than other food groups, such as legumes and nuts, Vitamin A-rich fruits and vegetables, when maternal education and household wealth improved. Dairy product consumption rose faster than other food groups when socioeconomic statuses, such as mothers’ education and household wealth, increased. The study also showed that the consumption of specific food items, food groups, and dietary diversity differ across Ghana’s 10 administrative regions. It was observed that consumption of grains, root, and tubers (88%) was relatively higher compared to Vitamin A-rich fruits and vegetables (12%) and legumes and nuts (13%) (Table 2). Based on this work’s findings, there is a need for nutritional policy interventions to improve child dietary diversity through proper infant and young child feeding practices to foster child development.

## Figures and Tables

**Table 1:** The eight food groups used for dietary diversity score calculation.

Food Groups	Food Items
1. Breastfeeding	Breast milk
2. Grains, roots and tubers	Soup/clear broth or bread, noodles, other grains or fortified baby food or potatoes, cassava, tubers
3. Legumes and nuts	Beans, peas and lentils
4. Dairy products	Formula milk or tinned powdered/fresh milk or cheese, yogurt, other milk products, or yogurt
5. Flesh foods	Liver, heart, other organ meat or fish, shellfish or chicken, duck or other birds,
6. Eggs	Eggs
7. Vitamin-A rich fruits and vegetables	Pumpkins, carrots, squash or dark green leafy vegetables or mangoes, papayas, vitamin A fruits
8. Other fruits and vegetables	Any other fruits

Source: WHO/UNICEF [28,58,59].

**Table 2:** Frequency and percentage of children by selected socio-demographic characteristics from the Ghana Demographic and Health Survey 2014 (*n* = 887).

Selected Characteristics	Frequency	Percentage
*Children characteristics*		
Age (months)		
6–11	293	33.1
23–12	594	66.9
Child’s sex		
Male	448	50.5
Female	439	49.5
Birth Order		
1	186	21
2	179	20.2
3	151	17
4+	371	41.8
*Mother characteristics*		
Mother’s education		
No education	250	28.2
Primary education	166	18.7
Secondary	437	49.3
Higher education	34	3.8
Ethnicity ^a^		
Akan	426	48.1
Ga/dangme	42	4.7
Ewe	112	12.6
guan	18	2.1
Mole-dagbani	163	18.4
Grusi	22	2.4
Gurma	68	7.7
Mande	13	1.5
Other	23	2.5
Region ^b^		
Western	89	10
Central	118	13.3
Greater Accra	128	14.5
Volta	65	7.3
Eastern	79	8.9
Ashanti	146	16.5
Bono-Ahafo	92	10.3
Northern	106	12
Upper east	41	4.6
Upper west	23	2.6
Religion		
Christianity	669	75.5
Islamic	163	18.4
African Traditionalist	26	2.9
Other/no religion	28	3.1
*Household characteristics*		
Wealth		
Poorest	201	22.7
Poorer	183	20.6
Middle	173	19.5
Richer	169	19
Richest	161	18.2
Place of residence		
Rural	414	46.7
Urban	473	53.3

^a^ Ethnicity: shows the major ethnics group in Ghana. ^b^ Region: represents 10 administrative regions of Ghana.

**Table 3:** Frequency and percentage of children’s consumed specific food items, food groups, dietary diversity from the Ghana Demographic and Health Survey 2014.

Selected Characteristics	Frequency	Percentage ^a^
*Specific food items* ^b^		
Juice	110	12.5
Tinned powdered/fresh milk	136	15.4
Fortified baby food	139	15.7
Other liquids	79	9
Bread, noodles, other grains	717	80.9
Potatoes, cassava, tubers	270	30.4
Eggs	180	20.4
Pumpkin, carrot, squash	61	7
Dark green leafy vegetables	322	36.4
Fruits	225	25.4
Meat (chicken, beef)	119	13.4
Liver, heart, other organ meat	26	3
Fish, shellfish	400	45.2
Beans, peas, lentils	114	12.9
Other solid/semi-liquid food	320	36.1
*Specific food groups* ^c^		
Breast milk	744	83.9
Grains, roots, and tubers	784	88.6
Legumes and nuts	114	12.9
Dairy products	197	22.2
Flesh foods	462	52.2
Eggs	180	20.4
Vitamin A-rich fruits and vegetables	106	12
Other fruits and vegetables	419	47.3
Dietary diversity score—mean (CI 95%)	3.39 (3.30–3.49)	
Dietary diversity consumption (DDC)		
Adequate	198	22.4
Inadequate	687	77.6

^a^ Percentage for the consumption of specific food items, specific food groups, and dietary diversity was calculated based on *N* = 887. ^b^ food items: specific food items consumed by the children during 24 h before the survey. ^c^ Food groups: specific food groups consumed by the children during 24 h before the survey.

**Table 4:** 24 h recall of consumption of food items, food groups and dietary diversity by mother’s education and household wealth of children aged 6–23 months, mean values (%) with confidence intervals (CIs), GDHS-2014.

*Food Consumption 24 h Recall*	Mother’s Education				Household Wealth				
	No Educ. (%)	Primary (%)	Secondary (%)	Higher (%)	Poorest (%)	Poorer (%)	Middle (%)	Richer (%)	Richest (%)
	(95% CI)	(95% CI)	(95% CI)	(95% CI)	(95% CI)	(95% CI)	(95% CI)	(95% CI)	(95% CI)
*Specific food items*									
Juice	8.8 (5.6–12.0)	9.3 (5.0–13.7)	13.8 (10.3–17.4)	37.2 (19.9–54.5)	3.5 (1.4–5.6)	10.6 (6.2–15.0)	10.6 (5.8–15.4)	15.1 (8.9–21.2)	25.1 (17.0–33.2)
Tinned powdered/fresh milk	5.9 (3.3–8.5)	11.3 (6.6–16.0)	19.6 (15.5–23.6)	50.3 (32.4–68.2)	5.7 (3.0–8.4)	9.6 (5.3–13.8)	8.9 (4.5–13.3)	21.8 (14.7–28.9)	34.2 (25.4–43.0)
Fortified baby food	4.0 (1.8–6.1)	6.6 (2.9–10.3)	21.9 (17.6–26.1)	66.2 (49.3–83.2)	2.7 (0.8–4.5)	4.8 (1.7–7.9)	10.0 (5.3–14.6)	25.0 (17.5–32.4)	40.6 (31.5–50.0)
Other liquids	13.9 (10.0–17.8)	10.0 (5.5–14.5)	6.2 (3.8–8.7)	2.6 (1.3–8.4)	14.9 (10.8–18.9)	6.1 (2.6–9.4)	7.6 (3.5–11.7)	11.0 (5.6–16.3)	4.3 (0.51–8.1)
Bread, noodles, other grains	80.7 (76.3–85.0)	82.1 (76.3–87.8)	80.0 (75.9–84.1)	87.6 (75.8–99.4)	79.7 (75.1–84.3)	77.2 (71.2–83.2)	82.2 (76.3–88.2)	81.0 (74.2–87.7)	84.9 (78.3–91.6)
Potatoes, cassava, tubers	33.8 (28.5–39.0)	30.0 (28.5–39.0)	28.0 (23.3–32.6)	39.8 (22.2–57.3)	29.8 (24.6–35.1)	37.1 (30.1–44.0)	29.0 (22.0–36.1)	20.9 (14.0–27.9)	35.1 (26.2–44.0)
Eggs	12.8 (9.1–16.6)	14.3 (9.1–19.5)	24.0 (19.6–28.3)	59.2 (41.6–76.8)	6.2 (3.5–9.0)	14.8 (9.7–19.8)	21.5 (15.2–27.9)	30.1 (22.2–38.0)	33.0 (24.3–41.8)
Pumpkin, carrot, squash	4.4 (2.1–6.7)	6.3 (2.6–10.0)	7.0 (4.4–9.6)	27.5 (11.5–43.5)	3.8 (1.6–6.0)	5.3 (2.1–8.5)	3.7 (0.7–6.6)	9.0 (4.1–13.9)	13.9 (7.5–20.4)
Dark green leafy vegetables	42.8 (37.4–48.3)	30.9 (23.9–37.8)	34.7 (29.8–39.5)	38.2 (20.8–55.6)	39.8 (34.2–45.5)	39.9 (32.9–46.9)	32.5 (25.2–39.8)	32.0 (24.0–40.0)	36.9 (27.9–45.8)
Fruits	20.1 (15.7–24.6)	22.2 (16.0–28.5)	26.9 (22.3–31.4)	59.8 (42.3–77.4)	11.9 (8.2–15.6)	20.8 (15.0–26.6)	26.3 (19.5–33.1)	34.7 (26.6–42.9)	36.7 (27.7–45.7)
Meat (chicken, beef)	13.6 (9.8–17.4)	9.8 (5.4–14.3)	14.1 (10.5–17.6)	21.3 (6.6–35.9)	9.5 (6.1–12.9)	11.7 (7.1–16.3)	13.7 (8.4–19.0)	15.9 (9.5–22.1)	17.510.4–24.5)
Liver, heart, other organ meat	2.6 (0.8–4.4)	0.7 (0.5–2.0)	2.9 (1.2–4.6)	17.7 (4.0–31.4)	1.1 (0.10–2.3)	5.0 (1.8–8.1)	2.1 (0.11–4.4)	1.6 (0.54–3.8)	5.3 (1.1–9.5)
Fish, shellfish	40.7 (35.2–46.1)	42.9 (35.5–50.3)	47.8 (42.7–52.9)	56.4 (38.6–74.1)	37.6 (32.0–43.1)	42.6 (35.6–49.7)	48.8 (41.0–56.6)	51.6 (43.1–60.2)	47.2 (37.9–56.5)
Beans, peas, lentils	15.5 (11.5–19.6)	12.7 (7.7–17.7)	11.2 (7.9–14.4)	15.6 (2.6–28.6)	15.7 (11.5–19.9)	10.6 (6.9–15.0)	5.6 (2.1–9.2)	17.4 (10.9–23.9)	15.0 (8.4–21.7)
Other solid/semi-liquid food	34.9 (29.6–40.2)	31.3 (24.4–38.3)	38.7 (33.7–43.7)	34.6 (17.6–51.7)	35.0 (29.5–40.5)	31.1 (24.4–37.7)	32.5 (25.2–39.8)	33.9 (25.8–42.0)	49.3 (40.0–58.6)
*Food groups*									
Breast milk	89.3 (85.9–92.8)	82.6 (77.0–88.3)	82.5 (78.6–88.3)	69.1 (52.5–85.6)	93.8 (91.0–96.6)	85.5 (75.9–87.0)	85.9 (80.5–91.3)	77.8 (70.8–84.9)	78.7 (71.0–86.3)
Grains, roots, and tubers	86.7 (82.9–90.4)	89.1 (84.4–93.8)	88.8 (85.5–92.0)	97.2 (91.2–100)	84.5 (80.3–88.6)	83.8 (78.5–89.1)	88.5 (83.6–93.5)	93.1 (88.7–97.4)	94.4 (90.1–98.7)
Legumes and nuts	15.5 (11.5–19.6)	12.7 (7.7–17.7)	11.2 (8.0–14.4)	15.6 (2.6–28.6)	15.7 (11.5–19.9)	10.6 (6.2–15.50)	5.6 (2.1–9.2)	17.4 (10.9–23.9)	15.0 (8.4–21.7)
Dairy products	11.3 (7.8–14.9)	15.7 (10.2–21.1)	27.8 (23.2–32.4)	62.6 (45.2–80.0)	6.3 (3.5–9.1)	13.6 (8.7–18.6)	13.5 (8.2–18.8)	31.2 (23.3–39.2)	52.1 (42.8–61.4)
Flesh foods	49.0 (43.7–54.6)	45.7 (38.2–53.2)	54.5 (49.4–59.6)	76.8 (61.7–91.9)	42.3 (36.6–48.0)	50.1 (43.0–57.3)	55.7 (48.0–63.4)	58.5 (50.0–66.9)	56.5 (47.3–65.7)
Eggs	12.8 (9.1–16.6)	14.3 (9.1–19.5)	24.0 (19.6–28.3)	59.2 (41.6–76.8)	6.2 (3.5–9.0)	14.8 (9.7–19.8)	21.5 (15.2–27.9)	30.1 (22.2–38.0)	33.0 (24.3–41.8)
Vit. A-rich fruits and vegetables	11.5 (7.9–15.0)	10.4 (5.8–15.0)	11.5 (8.2–14.8)	30.2 (13.8–46.7)	5.3 (2.7–7.8)	13.2 (8.4–18.1)	9.8 (5.2–14.4)	16.0 (9.7–22.3)	17.2 (10.1–24.2)
Other fruits and vegetables	49.8 (44.2–55.3)	39.5 (32.2–46.8)	46.8 (41.7–52.0)	72.7 (56.7–88.6)	43.6 (37.9–49.3)	45.0 (37.8–52.1)	41.7 (34.1–49.4)	51.4 (42.8–59.9)	56.2 (47.0–65.4)
Adequate dietary diversity consumption (ADDC)	18.8 (14.4–23.1)	14.1 (8.9–19.3)	24.2 (19.8–28.6)	65.1 (48.1–82.2)	12.8 (9.0–16.6)	16.1 (10.9–22.4)	16.9 (11.0–22.7)	31.0 (23.1–38.9)	38.3 (29.3–47.4)
Mean dietary diversity score	3.3 (3.1–3.4)	3.1 (2.9–3.3)	3.5 (3.3–3.6)	4.8 (4.2–5.4)	3.0 (2.8–3.1)	3.1 (2.9–3.3)	3.2 (3.0–3.4)	3.8 (3.5–4.0)	4.0 (3.7–4.3)

**Table 5:** 24 h recall of consumption of food items, food groups, and dietary diversity by geographical area of children aged 6–23 months, mean values (%) with confidence intervals (CIs), GDHS-2014.

*Food Consumption 24 h Recall*	Western	Central	Grt. Accra	Volta	Eastern	Ashanti	Bono-Ahafo	Northern	Upper East	Upper West
	(95% CI)	(95% CI)	(95% CI)	(95% CI)	(95% CI)	(95% CI)	(95% CI)	(95% CI)	(95% CI)	(95% CI)
*Specific food items*										
Juice	19.4 (10.8–28.0)	9.1 (3.4–14.8)	27.3 (16.6–38.0)	12.8 (4.6–21.1)	11.2 (4.2–18.3)	8.8 (2.6–15.1)	14.4 (7.7–21.1)	2.0 (0.3–4.4)	3.0 (0.5–6.6)	3.4 (0.7–7.6)
Tinned powdered/fresh milk	14.9 (7.1–22.7)	29.5 (20.6–38.5)	27.5 (16.8–38.2)	2.9 (1.2–7.0)	9.7 (3.1–16.3)	12.8 (5.4–20.0)	14.9 (8.1–21.7)	7.7 (3.2–12.1)	5.7 (0.9–10.7)	2.0 (0.1.1–5.2)
Fortified baby food	22.5 (13.4–31.6)	29.0 (20.1–37.9)	22.4 (12.4–32.4)	7.6 (1.1–14.2)	11.5 (4.4–18.6)	17.6 (9.2–25.9)	10.1 (4.3–15.7)	2.3 (−0.2–4.9)	9.8 (3.6–16.0)	3.1 (0.7–7.0)
Other liquids	8.8 (2.6–15.0)	3.7 (0.0–7.4)	11.5 (3.9–19.2)	6.7 (0.5–12.8)	10.5 (3.7–17.3)	3.9 (−0.3–8.1)	1.3 (−0.8–3.5)	27.0 (19.5–34.5)	5.1 (0.0–9.6)	10.0 (3.2–16.7)
Bread, noodles, other grains	86.5 (79.1–94.0)	82.7 (75.2–90.1)	88.8 (81.2–96.3)	82.5 (73.2–91.8)	66.3 (55.8–76.8)	73.5 (63.9–83.2)	83.0 (75.8–90.1)	81.6 (75.1–88.2)	84.6 (77.1–92.1)	79.7 (70.6–88.7)
Potatoes, cassava, tubers	44.3 (33.4–55.1)	32.2 (23.1–41.4)	40.2 (28.4–51.9)	16.9 (7.7–26.1)	20.9 (11.9–30.0)	13.0 (5.6–20.3)	47.1 (37.6–56.6)	36.6 (28.4–44.7)	12.1 (5.3–18.9)	32.9 (22.3–43.5)
Eggs	37.7 (27.1–48.3)	28.0 (19.2–36.8)	28.0 (19.2–36.8)	21.2 (11.1–31.2)	14.7 (6.8–22.5)	11.1 (4.2–18.1)	15.4 (8.5–22.3)	8.1 (3.5–12.7)	4.3 (0.0–8.5)	15.5 (7.4–23.7)
Pumpkin, carrot, squash	6.6 (1.1–12.0)	11.0 (4.8–17.1)	15.1 (6.5–23.7)	2.4 (1.3–6.1)	5.8 (0.6–10.9)	2.2 (1.0–5.5)	7.1 (2.2–12.0)	3.7 (0.5–6.9)	0.6 (−0.9–2.2)	12.8 (5.3–20.3)
Dark green leafy vegetables	51.1 (40.2–62.0)	25.8 (17.2–34.4)	42.3 (30.4–54.1)	42.7 (30.6–54.9)	21.0 (11.9–30.0)	26.7 (17.0–36.4)	46.9 (37.4–56.5)	31.3 (23.5–39.2)	50.2 (39.8–60.6)	53.6 (42.4–64.8)
Any other fruits	36.4 (25.9–46.9)	37.8 (28.3–47.4)	43.9 (32.0–55.8)	15.7 (6.8–24.6)	8.1 (2.0–14.1)	9.7 (3.2–16.2)	12.7 (6.4–19.1)	13.1 (7.4–18.8)	6.9 (1.6–12.1)	9.1 (2.7–15.6)
Meat (chicken, beef)	10.1 (3.5–16.7)	9.6 (3.8–15.4)	29.8 (18.8–40.8)	11.5 (3.719.3)	5.5 (0.4–10.6)	9.2 (2.8–15.5)	14.6 (7.8–21.3)	13.9 (8.1–19.8)	13.0 (6.0–20.0)	7.9 (1.8–13.9)
Liver, heart, other organ meat	5.9 (0.8–11.1)	2.0 (−0.7–4.7)	2.5 (−1.2–6.2)	3.3 (−1.1–7.6)	1.1 (−1.2–3.3)	1.8 (−1.1–4.7)	5.1 (0.9–9.2)	3.9 (0.6–7.2)	1.8 (−1.0–4.6)	1.2 (−1.2–3.7)
Fish, shellfish	44.3 (33.4–55.1)	66.4 (57.1–75.7)	58.7 (46.9–70.5)	39.8 (27.8–518)	30.3 (20.1–40.5)	33.4 (23.1–43.8)	57.0 (47.6–66.5)	38.9 (30.6–47.1)	19.3 (11.1–27.5)	36.2 (25.4–47.1)
Beans, peas, lentils	8.7 (2.6–14.9)	8.4 (3.0–14.0)	28.4 (17.6–39.2)	5.6 (0.0–11.2)	9.4 (2.9–15.9)	3.0 (0.7–6.8)	13.6 (7.1–20.1)	18.4 (11.8–24.9)	15.7 (8.2–23.3)	25.4 (15.6–35.2)
Other solid/semi-liquid food	29.9 (19.9–39.9)	15.4 (8.3–22.5)	60.5 (48.8–72.3)	13.0 (4.8–21.3)	27.1 (17.2–37.0)	38.8 (28.1–49.5)	63.3 (54.1–72.5)	38.6 (30.4–46.8)	11.5 (4.8–18.1)	30.1 (19.7–40.4)
*Food groups*										
Breast milk	73.5 (63.8–83.1)	77.5 (69.3–85.6)	77.9 (68.0–87.9)	89.5 (82.0–97.0)	76.3 (66.8–85.7)	91.1 (84.9–98.7)	83.1 (75.9–90.2)	95.1 (91.4–98.7)	91.0 (85.1–97.0)	94.8 (89.9–100.0)
Grains, roots, and tubers	91.1 (84.9–97.3)	93.8 (89.1–98.5)	96.6 (92.3–100)	90.9 (83.8–97.9)	79.1 (70.1–88.2)	82.6 (74.3–90.9)	90.9 (85.5–96.4)	83.6 (77.3–89.8)	87.3 (80.4–94.2)	87.0 (79.4–94.5)
Legumes and nuts	8.7 (2.6–14.9)	8.5 (3.0–14.0)	28.4 (17.6–39.2)	5.6 (0.0–11.2)	9.4 (2.9–15.9)	3.0 (−0.7–6.8)	13.6 (7.1–20.1)	18.4 (11.8–24.9)	15.7 (8.1–23.3)	25.4 (15.6–35.2)
Dairy products	22.8 (13.6–31.9)	33.0 (23.7–42.2)	54.9 (43.0–66.9)	5.1 (−0.2–10.6)	18.3 (9.7–26.9)	15.3 (7.4–23.2)	16.6 (9.5–23.7)	8.4 (3.7–13.1)	6.3 (1.3–11.4)	4.1 (−0.4–8.5)
Flesh foods	46.6 (35.8–57.5)	72.0 (63.2–80.8)	69.4 (58.4–80.5)	49.3 (37.0–61.5)	35.0 (24.4–45.6)	40.3 (29.5–51.0)	64.4 (55.2–73.5)	46.2 (37.7–54.6)	30.2 (20.6–39.7)	38.6 (27.7–49.6)
Eggs	37.7 (27.1–48.3)	28.0 (19.2–36.8)	34.9 (23.4–46.3)	21.1 (11.1–31.2)	14.7 (6.8–22.5)	11.1 (4.2–18.1)	15.4 (8.5–22.3)	8.1 (3.4–12.7)	4.3 (0.8–8.5)	15.5 (7.4–23.7)
Vit. A-rich fruits and vegetables	16.0 (8.0–24.0)	17.2 (9.8–24.6)	20.6 (10.9–30.3)	8.7 (1.8–15.7)	10.7 (3.8–17.5)	6.0 (0.8–11.3)	14.8 (8.1–21.6)	5.1 (1.4–8.9)	0.6 (−0.1–2.2)	14.3 (6.3–22.1)
Other fruits and vegetables	61.4 (50.7–72.0)	52.0 (42.2–61.8)	67.2 (56.0–78.5)	45.1 (32.9–57.3)	25.7 (16.0–35.4)	29.7 (19.7–39.7)	52.4 (42.9–61.9)	39.3 (31.1–47.6)	53.4 (43.0–63.8)	55.3 (44.1–66.5)
Adequate dietary diversity consumption (ADDC)	23.5 (14.3–32.8)	33.2 (24.0–42.4)	49.4 (37.4–61.4)	14.6 (5.9–23.2)	10.9 (4.0–17.8)	6.7 (1.2–12.2)	25.8 (17.5–34.1)	13.8 (8.0–19.6)	9.2 (3.2–15.3)	22.1 (12.7–31.6)
Mean Dietary diversity score	3.6 (3.3–3.9)	3.8 (3.6–4.1)	4.5 (4.14.9)	3.2 (2.9–3.4)	2.7 (2.4–3.0)	2.8 (2.5–3.1)	3.5 (3.2–3.8)	3.0 (2.8–3.3)	2.9 (2.7–3.1)	3.3 (3.0–3.6)

**Table 6:** The association between household wealth and consumption of food items, food groups, and adequate dietary diversity of children aged 6–23 months, adjusted odds ratios (ORs) with confidence intervals (Cis), GDHS-2014.

*Food Consumption 24 h Recall*	Model A				Model B			
	Poorer OR (95%)	Middle OR (95%)	Richer (95% CI)	Richest (95% CI)	Poorer (95% CI)	Middle (95% CI)	Richer (95% CI)	Richest (95% CI)
*Specific food items*								
Juice	2.95 (1.28–6.85)	3.97 (1.72–9.17)	5.74 (2.53–13.00)	12.27 (5.59–26.93)	2.38 (0.95–5.95)	2.62 (0.99–6.90)	3.35 (1.16–9.70)	5.46 (1.70–17.50)
Tinned powdered/fresh milk	1.50 (0.71–3.19)	1.94 (0.92–4.08)	5.24 (2.70–10.18)	10.44 (5.45–19.98)	0.98 (0.43–2.25)	1.12 (0.46–2.69)	2.87 (1.15–7.12)	4.90 (1.76–13.68)
Fortified baby food	2.00 (0.77–5.19)	4.41 (1.88–10.36)	11.54 (5.12–26.00)	24.00 (10.72–53.70)	1.67 (0.59–4.70)	3.69 (1.34–10.13)	11.60 (3.93–34.21)	21.18 (6.33–70.76)
Other liquids	0.53 (0.27–1.02)	0.72 (0.38–1.37)	0.71 (0.36–1.42)	0.47 (0.20–1.09)	0.79 (0.38–1.64)	1.08 (0.48–2.42)	1.25 (0.47–3.36)	0.85 (0.24–3.00)
Bread, noodles, other grains	0.71 (0.45–1.13)	1.08 (0.65–1.78)	0.90 (0.53–1.53)	1.14 (0.63–2.06)	0.71 (0.42–1.19)	1.14 (0.62–2.09)	1.03 (0.51–2.09)	1.49 (0.63–3.51)
Potatoes, cassava, tubers	1.28 (0.86–1.91)	0.81 (0.52–1.26)	0.54 (0.32–0.89)	1.16 (0.72–1.86)	1.31 (0.82–2.09)	0.89 (0.50–1.55)	0.52 (0.26–1.05)	1.01 (0.47–2.17)
Eggs	2.58 (1.39–4.79)	4.70 (2.57–8.60)	4.43 (2.37–8.27)	7.46 (4.02–13.83)	2.39 (1.20–4.76)	4.10 (1.10–8.58)	2.36 (1.58–8.67)	3.00 (1.96–13.06)
Pumpkin, carrot, squash	1.06 (0.46–2.42)	1.22 (0.52–2.87)	1.55 (0.67–3.56)	3.42 (1.63–7.18)	1.02 (0.40–2.61)	1.33 (0.47–3.75)	1.78 (0.54–5.86)	4.02 (1.03–15.64)
Dark green leafy vegetables	1.01 (0.69–1.48)	0.77 (0.51–1.16)	0.78 (0.51–1.20)	0.89 (0.57–1.41)	1.05 (0.68–1.61)	0.77 (0.47–1.28)	0.80 (0.44–1.46)	0.87 (0.43–1.75)
Fruits	2.00 (1.19–3.21)	2.56 (1.54–4.26)	3.05 (1.81–5.11)	3.96 (2.34–6.73)	1.88 (1.07–3.30)	2.66 (1.42–5.02)	3.54 (1.69–7.40)	3.97 (1.70–9.29)
Meat (chicken, beef)	1.17 (0.66–2.07)	1.16 (9.63–2.15)	1.78 (0.98–3.20)	1.83 (0.99–3.37)	1.25 (0.65–2.39)	1.08 (0.51–2.31)	1.31 (0.55–3.08)	1.25 (0.47–3.36)
Liver, heart, other organ meat	4.07 (1.25–13.23)	2.00 (0.49–8.17)	2.29 (0.56–9.34)	5.53 (1.62–18.85)	3.78 (1.04–13.76)	2.06 (0.41–10.29)	2.17 (0.34–13.81)	3.46 (0.45–26.64)
Fish, shellfish	1.33 (0.91–1.96)	1.43 (0.96–2.16)	1.41 (0.92–2.17)	1.45 (0.92–2.28)	1.13 (0.73–1.75)	01.23 (0.74–2.04)	1.18 (0.65–2.14)	1.19 (0.59–2.40)
Beans, peas, lentils	0.73 (0.43–1.23)	0.41 (0.21–0.80)	0.87 (0.49–1.55)	0.90 (0.50–1.63)	0.72 (0.40–1.30)	0.37 (0.17–0.82)	0.71 (0.31–1.62)	0.67 (0.25–1.75)
Other solid/semi-liquid food	0.81 (0.55–1.21)	0.81 (0.53–1.24)	0.93 (0.60–1.44)	1.77 (1.13–2.76)	0.71 (0.45–1.12)	0.61 (0.36–1.02)	0.58 (0.31–1.08)	1.11 (0.54–2.26)
*Food groups*								
Breast milk	0.30 (0.16–0.55)	0.28 (0.14–0.54)	0.17 (0.09–0.33)	0.18 (0.10–0.36)	0.31 (0.15–0.65)	0.26 (0.11–0.59)	0.17 (0.07–43.5)	0.20 (0.07–0.57)
Grains, roots, and tubers	0.73 (0.44–1.20)	1.25 (0.70–2.21)	1.61 (0.83–3.15)	2.19 (1.00–4.88)	0.66 (0.38–1.16)	1.29 (0.65–2.57)	1.90 (0.81–4.46)	2.62 (0.91–7.53)
Legumes and nuts	0.73 (0.43–1.23)	0.41 (0.21–0.80)	0.87 (0.49–1.5)	0.90 (0.50–1.63)	0.72 (0.40–1.30)	0.37 (0.17–0.82)	0.70 (0.31–1.62)	0.67 (0.25–1.75)
Dairy products	1.96 (1.00–3.84)	2.62 (1.34–5.10)	7.64 (4.14–14.09)	18.04 (9.72–33.47)	1.37 (0.65–2.88)	1.59 (0.72–3.51)	4.17 (1.82–9.57)	8.00 (3.12–20.29)
Flesh foods	1.45 (0.98–2.13)	1.60 (1.06–2.41)	1.61 (1.04–2.49)	1.88 (1.18–2.10)	1.29 (0.83–2.00)	1.31 (0.79–2.18)	1.20 (0.66–2.18)	1.26 (0.62–2.56)
Eggs	2.58 (1.39–4.79)	4.70 (2.57–8.60)	4.43 (2.37–8.27)	7.45 (4.02–13.83)	2.39 (1.20–4.76)	4.10 (1.96–8.58)	3.71 (1.58–8.8.67)	5.06 (1.96–13.06)
Vit. A-rich fruits and vegetables	1.94 (1.03–3.65)	1.90 (0.97–3.74)	2.51 (1.29–4.87)	3.42 (1.77–6.60)	1.94 (0.94–4.02)	2.05 (0.89–4.71)	2.70 (1.05–6.98)	3.59 (1.11–10.78)
Other fruits and vegetables	1.10 (0.76–1.61)	1.04 (0.69–1.55)	1.12 (0.73–1.71)	1.50 (0.95–2.35)	1.17 (0.76–1.80)	1.08 (0.66–1.77)	1.26 (0.70–2.27)	1.51 (0.75–3.03)
Adequate dietary diversity consumption (ADDC)	1.25 (0.75–2.07)	1.46 (0.86–2.48)	1.97 (1.17–3.32)	3.49 (2.08–5.85)	1.13 (0.64–2.00)	1.31 (0.69–2.49)	1.67 (0.80–3.51)	2.53 (1.09–5.90)

Note: Model A adjusted child’s age and gender, Model B adjusted full characteristics (child’s age, gender, place of residence, religion, ethnicity, birth order, and mother’s education). Poorest was chosen as the reference category in all the models.

**Table 7:** The association between mother’s education and consumption of food items, food groups, and adequate dietary diversity of children aged 6–23 months, adjusted odds ratios (ORs) with confidence intervals (CIs), GDHS-2014.

*Food Consumption 24 h Recall*	Model A			Model B		
	Primary OR (95% CI)	Secondary OR (95% CI)	Higher OR (95% CI)	Primary OR (95% CI)	Secondary OR (95% CI)	Higher OR (95% CI)
*Specific food items*						
Juice	0.97 (0.44–2.13)	2.72 (1.57–4.71)	7.54 (3.09–18.41)	0.52 (0.22–1.26)	1.00 (0.47–2.06)	2.07 (0.66–6.50)
Tinned powdered/fresh milk	2.17 (1.08–4.38)	4.24 (2.40–7.49)	10.45 (4.27–25.56)	1.43 (0.66–3.12)	1.61 (0.77–3.36)	2.22 (0.73–6.78)
Fortified baby food	2.17 (0.98–4.82)	6.00 (3.18–11.31)	27.34 (10.87–68.78)	1.43 (0.60–3.46)	2.07 (0.93–4.61)	4.93 (1.60–15.17)
Other liquids	1.07 (0.60–1.93)	0.60 (0.35–1.03)	1.19 (0.39–3.63)	1.97 (1.00–3.86)	1.27 (0.61–2.65)	2.81 (0.68–11.67)
Bread, noodles, other grains	0.92 (0.58–1.49)	0.99 (0.67–1.45)	0.65 (0.27–1.58)	0.91 (0.54–1.56)	0.83 (0.49–1.41)	0.66 (0.17–1.39)
Potatoes, cassava, tubers	0.85 (0.56–1.29)	0.93 (0.66–1.30)	0.99 (0.44–2.23)	0.81 (0.49–1.33)	0.86 (0.53–1.40)	1.09 (0.40–3.00)
Eggs	1.04 (0.57–1.89)	2.57 (1.67–3.98)	5.47 (2.40–12.47)	0.68 (0.35–1.34)	1.18 (0.65–2.14)	1.95 (0.70–5.42)
Pumpkin, carrot, squash	2.10 (1.00–4.37)	1.36 (0.70–2.67)	6.20 (2.26–16.98)	1.85 (0.80–4.27)	0.93 (0.37–2.33)	3.06 (0.76–12.37)
Dark green leafy vegetables	0.60 (0.41–0.90)	0.88 (0.65–1.21)	0.83 (0.38–1.79)	0.62 (0.40–0.98)	1.09 (0.71–1.69)	1.11 (0.45–2.75)
fruits	1.40 (0.85–2.29)	2.23 (1.51–3.29)	4.16 (1.87–9.24)	1.00 (0.57–1.75)	1.26 (0.74–2.14)	2.00 (0.78–5.54)
Meat (chicken, beef)	0.91 (0.51–1.65)	1.34 (0.86–2.11)	1.68 (0.64–4.44)	1.09 (0.56–2.13)	1.55 (0.81–2.96)	1.85 (0.55–6.22)
Liver, heart, other organ meat	0.79 (0.29–3.10)	1.78 (0.71–4.47)	10.75 (3.30–35.00)	0.54 (0.12–2.39)	1.42 (0.40–5.09)	12.46 (1.94–80.01)
Fish, shellfish	1.10 (0.75–1.64)	1.49 (1.08–2.05)	0.98 (0.45–2.13)	0.98 (0.32–2.08)	1.18 (0.76–1.83)	0.82 (0.32–2.08)
Beans, peas, lentils	0.98 (0.57–1.66)	0.89 (0.57–1.37)	0.81 (0.27–2.45)	1.29 (0.70–2.36)	1.22 (0.66–2.26)	1.16 (0.31–4.34)
Other solid/semi-liquid food	0.86 (0.58–1.29)	1.21 (0.88–1.66)	0.65 (0.28–1.52)	0.83 (0.53–1.33)	0.92 (0.58–1.45)	0.36 (0.13–0.98)
*Food groups*						
Breast milk	0.56 (0.31–0.99)	0.43 (0.27–0.69)	0.18 (0.07–0.43)	1.14 (0.58–2.27)	1.33 (0.69–2.55)	0.68 (0.21–2.15)
Grains, roots, and tubers	1.09 (0.63–1.88)	1.20 (0.77–1.88)	1.53 (0.44–5.34)	0.93 (0.51–1.72)	0.78 (0.42–1.45)	0.85 (0.20–3.56)
Legumes and nuts	0.98 (0.58–1.66)	0.89 (0.57–1.37)	0.81 (0.27–2.45)	1.29 (0.70–2.36)	1.22 (0.66–2.26)	1.15 (0.31–4.34)
Dairy products	1.97 (1.08–3.59)	4.29 (2.66–6.92)	11.84 (5.20–26.96)	1.25 (0.63–2.49)	1.46 (0.77–2.77)	2.26 (0.81–6.27)
Flesh foods	0.98 (0.66–1.46)	1.52 (1.11–2.09)	1.90 (0.86–4.20)	0.85 (0.55–1.33)	1.14 (0.73–1.78)	1.44 (0.56–3.69)
Eggs	1.04 (0.57–1.89)	2.57 (1.67–3.98)	5.47 (2.40–12.47)	0.68 (0.35–1.34)	1.18 (0.65–2.14)	1.95 (0.70–5.42)
Vit. A-rich fruits and vegetables	1.42 (0.79–2.55)	1.28 (0.78–2.11)	3.42 (1.39–8.45)	0.92 (0.47–1.82)	0.68 (0.34–1.35)	1.65 (0.51–5.34)
Other fruits and vegetables	0.67 (0.45–0.98)	1.10 (0.81–1.50)	1.74 (0.80–3.79)	0.64 (0.41–1.00)	1.07 (0.70–1.65)	1.62 (0.65–4.02)
Adequate dietary diversity consumption (ADDC)	0.94 (0.56–1.58)	1.63 (1.10–2.41)	3.52 (1.58–7.85)	0.84 (0.47–1.52)	1.21 (0.70–2.10)	2.18 (0.80–5.92)

Note: Model A adjusted child’s age and gender, Model B adjusted full characteristics (child’s age, gender, place of residence, religion, ethnicity, birth order, and wealth). No education was chosen as the reference category in all the models.

**Table 8:** Average Predictive Margins (APMs) with standard error (SE) for a geographical area on consuming specific food items and food groups among children aged 6–23 months, GDHS-2014.

*Food Consumption 24 h Recall*	Western	Central	Grt. Accra	Volta	Eastern	Ashanti	Bono–Ahafo	Northern	Upper East	Upper West
	(SE)	(SE)	(SE)	(SE)	(SE)	(SE)	(SE)	(SE)	(SE)	(SE)
*Specific food items*										
Juice	0.18 (0.042)	0.14 (0.039)	0.18 (0.047)	0.14 (0.059)	0.10 (0.033)	0.07 (0.026)	0.13 (0.034)	0.04 (0.019)	0.06 (0.037)	0.05 (0.029)
Tinned powdered/fresh milk	0.12 (0.033)	0.21 (0.041)	0.13 (0.031)	0.03 (0.025)	0.07 (0.235)	0.11 (0.0293)	0.18 (0.037)	0.18 (0.057)	0.16 (0.061)	0.04 (0.032)
Fortified baby food	0.18 (0.037)	0.19 (0.036)	0.13 (0.030)	0.16 (0.059)	0.11 (0.031)	0.10 (0.024)	0.15 (0.034)	0.04 (0.026)	0.17 (0.057)	0.05 (0.029)
Other liquids	0.10 (0.037)	0.08 (0.037)	0.17 (0.066)	0.12 (0.058)	0.12 (0.049)	0.05 (0.027)	0.02 (0.013)	0.18 (0.041)	0.04 (0.019)	0.09 (0.030)
Bread, noodles, other grains	0.82 (0.044)	0.82 (0.044)	0.80 (0.061)	0.78 (0.067)	0.63 (0.062)	0.73 (0.052)	0.79 (0.040)	0.84 (0.038)	0.84 (0.042)	0.82 (0.047)
Potatoes, cassava, tubers	0.43 (0.054)	0.40 (0.054)	0.42 (0.068)	0.16 (0.051)	0.20 (0.047)	0.16 (0.043)	0.45 (0.046)	0.30 (0.053)	0.15 (0.044)	0.24 (0.556)
Eggs	0.33 (0.050)	0.19 (0.040)	0.23 (0.051)	0.19 (0.059)	0.12 (0.037)	0.10 (0.030)	0.13 (0.033)	0.11 (0.039)	0.12 (0.050)	0.17 (0.058)
Pumpkin, carrot, squash	0.08 (0.035)	0.17 (0.053)	0.14 (0.047)	0.02 (0.016)	0.04 (0.023)	0.04 (0.028)	0.11 (0.035)	0.08 (0.041)	0.01 (0.012)	0.13 (0.048)
Dark green leafy vegetables	0.51 (0.057)	0.41 (0.057)	0.46 (0.070)	0.41 (0.079)	0.27 (0.056)	0.31 (0.056)	0.46 (0.049)	0.28 (0.050)	0.40 (0.062)	0.470 (0.067)
fruits	0.32 (0.052)	0.33 (0.056)	0.36 (0.066)	0.11 (0.041)	0.08 (0.030)	0.12 (0.038)	0.17 (0.038)	0.17 (0.046)	0.10 (0.039)	0.12 (0.044)
Meat (chicken, beef)	0.08 (0.030)	0.14 (0.040)	0.23 (0.058)	0.15 (0.059)	0.07 (0.033)	0.08 (0.030)	0.18 (0.038)	0.13 (0.035)	0.17 (0.050)	0.11 (0.042)
Liver, heart, other organ meat	0.08 (0.030)	0.03 (0.018)	0.01 (0.011)	0.03 (0.025)	0.01 (0.008)	0.02 (0.017)	0.08 (0.030)	0.10 (0.052)	0.07 (0.052)	0.05 (0.037)
Fish, shellfish	0.45 (0.055)	0.65 (0.052)	0.60 (0.066)	0.36 (0.074)	0.34 (0.058)	0.37 (0.057)	0.53 (0.047)	0.40 (0.057)	0.21 (0.049)	0.27 (0.056)
Beans, peas, lentils	0.10 (0.033)	0.12 (0.035)	0.23 (0.058)	0.03 (0.014)	0.08 (0.027)	0.05 (0.028)	0.13 (0.034)	0.23 (0.061)	0.26 (0.071)	0.30 (0.075)
Other solid/semi-liquid food	0.31 (0.053)	0.22 (0.045)	0.53 (0.072)	0.14 (0.049)	0.26 (0.053)	0.43 (0.059)	0.61 (0.047)	0.39 (0.060)	0.13 (0.045)	0.36 (0.069)
*Food groups*										
Breast milk	0.75 (0.042)	0.82 (0.036)	0.87 (0.036)	0.89 (0.045)	0.80 (0.044)	0.89 (0.032)	0.86 (0.032)	0.91 (0.039)	0.82 (0.061)	0.85 (0.056)
Grains, roots, and tubers	0.89 (0.036)	0.91 (0.032)	0.92 (0.044)	0.88 (0.052)	0.76 (0.060)	0.83 (0.045)	0.90 (0.029)	0.86 (0.038)	0.87 (0.040)	0.83 (0.048)
Legumes and nuts	0.10 (0.033)	0.12 (0.035)	0.23 (0.058)	0.03 (0.014)	0.09 (0.027)	0.05 (0.028)	0.13 (0.034)	0.23 (0.061)	0.26 (0.071)	0.30 (0.075)
Dairy products	0.20 (0.039)	0.25 (0.042)	0.27 (0.050)	0.08 (0.039)	0.13 (0.035)	0.13 (0.033)	0.13 (0.030)	0.18 (0.051)	0.20 (0.064)	0.09 (0.045)
Flesh foods	0.47 (0.054)	0.74 (0.046)	0.67 (0.063)	0.46 (0.077)	0.40 (0.060)	0.44 (0.056)	0.63 (0.045)	0.47 (0.057)	0.32 (0.058)	0.30 (0.057)
Eggs	0.33 (0.050)	0.19 (0.043)	0.23 (0.051)	0.19 (0.059)	0.12 (0.037)	0.10 (0.030)	0.13 (0.033)	0.11 (0.039)	0.12 (0.050)	0.17 (0.058)
Vit. A-rich fruits and vegetables	0.16 (0.042)	0.25 (0.054)	0.22 (0.059)	0.06 (0.032)	0.10 (0.038)	0.09 (0.037)	0.21 (0.042)	0.10 (0.040)	0.01 (0.010)	0.12 (0.039)
Other fruits and vegetables	0.60 (0.055)	0.55 (0.056)	0.65 (0.066)	0.43 (0.078)	0.31 (0.058)	0.34 (0.056)	0.53 (0.048)	0.38 (0.055)	0.48 (0.064)	0.51 (0.067)
Adequate dietary diversity consumption (ADDC)	0.23 (0.047)	0.32 (0.056)	0.43 (0.074)	0.10 (0.040)	0.11 (0.036)	0.08 (0.033)	0.30 (0.047)	0.14 (0.040)	0.14 (0.046)	0.18 (0.051)

## Data Availability

Restrictions apply to the availability of these data. Data was obtained from demographic and health survey (DHS) and are available at https://dhsprogram.com/data/available-datasets.cfm (accessed on 11 February 2021) with the permission of DHS.

## Appendix A

**Table A1:** Dietary diversity intake in the last 24 h by Ghanaian children aged 24–59 months, GDHS-14 (*N* = 534).

Characteristics	Frequency	Percentage
Dietary diversity intake		
Adequate	68	12.8
Inadequate	466	87.2
Dietary diversity score—mean (CI 95%)	2.63 (2.29–2.77)	

**Table A2:** The association between mother’s education, household and adequate dietary diversity of children aged 24–59 months, adjusted odds ratios (ORs) with confidence intervals (CIs), GDHS-2014.

Characteristics	Frequency	Percentage
Dietary diversity intake		
Adequate	68	12.8
Inadequate	466	87.2
Dietary diversity score—mean (CI 95%)		2.63 (2.29–2.77)
